# A targeted one dimensional fully convolutional autoencoder network for intelligent compression of magnetic flux leakage data

**DOI:** 10.1038/s41598-025-96282-2

**Published:** 2025-04-03

**Authors:** Wenbo Xuan, Pengchao Chen, Rui Li, Fuxiang Wang, Kuan Fu, Zhitao Wen

**Affiliations:** 1 PipeChina Institute of Science and Technology, No. 4 Dongting 1st Street, Tianjin Economic-Technological Development Area, Tianjin, 300457 China; 2 Northeastern University, No. 11, Lane 3, Culture Road, Heping District, Shenyang, Liaoning Province China

**Keywords:** Electrical and electronic engineering, Mechanical engineering

## Abstract

In response to the issue of massive data volume generated by magnetic flux leakage (MFL) non-destructive testing in oil and gas pipelines, an intelligent data compression method based on a targeted one-dimensional fully convolutional autoencoder network is proposed. Firstly, a data preprocessing module is designed to generate high-quality data required for subsequent processing, taking into account the characteristics of MFL data. Secondly, a data block classification algorithm is developed to calculate peak values for segmented differential data, and based on a predefined targeted threshold, distinguish different types of MFL data. Subsequently, based on the distinct data types, targeted one-dimensional fully convolutional autoencoder models are constructed to effectively achieve dimensionality reduction compression and reconstruction of the MFL data. Through practical experimental analysis, the reconstruction error such as MAE is reduced by about 27.7% and the compression ratio is improved by about 14% compared with traditional methods such as PCA. In addition, compared with ID-AE, the proposed 1D-FCAE reduces 206.8 k, 1.58G, and 80 s in parameters, memory usage, and training time, respectively, and reduces compression and decompression time by 60 ms and 69 ms, respectively, validating that it is easy to be applied in industrial environments with limited resources.

## Introduction

Magnetic flux leakage (MFL) is one of the most commonly used methods for detecting defects in long-distance oil and gas pipelines^1–4^. With the development of sensing electronic technology, MFL inspection has made significant progress, enabling ultra-high-speed acquisition and large-capacity storage with internal detectors. However, as the number of circumferential sensors and axial sampling frequency increases, the volume of MFL inspection data grows dramatically, reaching several terabytes per inspection, posing significant challenges to data analysis and storage^5^. In order to improve data analysis efficiency, reduce data storage space, and facilitate data management, it is necessary to compress the MFL data^6^. Therefore, the development of a non-destructive compression method holds crucial importance for the analysis of MFL data in long-distance oil and gas pipelines.

Common data compression algorithms include wavelet transform coding^7^, LZW (Lempel Ziv Welch) algorithm^8,9^, and Huffman algorithm^10^, among others. In recent years, scholars have conducted in-depth research on compression methods for MFL data. For instance, Zhang^5^ addressed the contradiction between compression performance and data reconstruction effectiveness by designing an MFL data compression method based on adaptive compressive sensing. Wang et al.^11^ tackled the issue of storing and analyzing massive amounts of high-definition MFL inspection data for pipelines and proposed a feature lossless compression method based on combined filtering. Zhang et al.^12^ combined compressive sensing with important segment selection to propose an online compressive sensing method for MFL inspection data. Song et al.^13^ researched a denoising and compression algorithm for MFL detection signals based on wavelet packet-Haar wavelet algorithm, which preserves the characteristics of high-frequency signal components, avoiding signal distortion phenomena during decompression.

In summary, existing MFL data compression methods achieve data compression by eliminating redundancy based on the characteristics of MFL data. However, such methods usually require the design of complex compression and decompression encoding algorithms, making them time-consuming and challenging to operate in industrial environments. In addition, these compression methods require extensive hyper-parameter tuning and setup by domain experts, which not only further reduces their operational efficiency but also results in very limited generalizability for practical industrial applications.

With the development of artificial intelligence, data compression methods based on shallow machine learning gradually emerged. Some classical machine learning dimensionality reduction methods such as PCA^14^ and KPCA^15^ have been widely applied. However, MFL signals possess strong nonlinearities and the internal feature variations of different types of signals, such as defects and flanges, possess strong complexity, which makes it difficult for shallow machine learning methods to effectively learn the deeper representation of signals. In contrast, deep learning demonstrates strong automatic feature learning capabilities using mechanisms such as gradient optimization and backpropagation, enabling it to learn the intrinsic deep features of complex MFL signals^16,17^.

Among deep learning techniques, Transformer^18^, diffusion models^19^, and autoencoders^20^ have achieved great success in time series processing tasks. For time series data compression, autoencoders have been widely applied by researchers due to their unsupervised learning characteristics and have yielded significant results^21–23^. Autoencoders aim to map signals from a high-dimensional space to a low-dimensional feature space through an encoder to obtain latent representations, and then reconstruct the original signal through a decoder, thereby achieving compression and decompression. Sheena et al.^24^ proposed a genome sequence compression method called GenCoder based on a convolutional autoencoder. Xue et al.^24^ introduced frequency domain information into the variational autoencoder and proposed a frequency-enhanced vector quantized variational autoencoder (FEVQVAE) method to compress structural vibration response signals with a higher compression ratio. Song et al.^25^ proposed a compression method that combines a deep encoder with Huffman coding for wellbore trajectory data compression. Although these methods effectively utilize autoencoders to address compression tasks, the complexity of MFL signals may hinder the direct transfer of these methods in precisely restoring signal details. Moreover, their complex structures often introduce a large number of parameters and computational overhead, which may be unfavorable for online applications in industrial environments.

Therefore, considering the aforementioned factors, this paper introduces a convolutional neural network (CNN) into the autoencoder structure and proposes a lightweight one-dimensional fully convolutional autoencoder (ID-FCAE) architecture. The pooling layers are replaced with downsampled convolutional layers to enhance the model’s feature extraction capability, and the lightweight design facilitates deployment in industrial site. Additionally, recognizing the data correlation in the autoencoder model, data preprocessing and data classification modules are designed at the front end of the model. Depending on different data types, targeted ID-FCAE models are constructed, significantly improving the model’s generalization performance and enabling efficient dimensionality reduction compression of MFL signals.

The contribution of this paper focuses on two aspects:

1. A front-end data preprocessing and classification module is proposed, in which a data preprocessing algorithm is developed to improve the quality of MFL signals based on the MFL signal characteristics, while a window length is set in the classification module to acquire data blocks and a refinement threshold is set to discriminate the types of the post-differential data blocks, which provides distinguishable samples for the back-end model construction.
2. Based on the distinguishable samples, in the back-end, the targeted fully convolutional autoencoder (ID-FCAE) architecture is proposed, in which the convolutional neural network is introduced into the autoencoder structure, and the downsampled convolutional layer is used to replace the traditional pooling layer, which greatly improves the feature extraction capability and significantly reduces the model complexity, making it more suitable for online applications in the industrial field.

The remainder of the paper is organized as follows: Section “Methodology” introduces the proposed method in detail. Section “Experimental validation” carries out the experimental validation and discusses the results. Section “Conclusion” summarizes the whole paper and provides future research directions.

## Methodology

### Overall scheme of MFL data compression

**Fig. 1:**
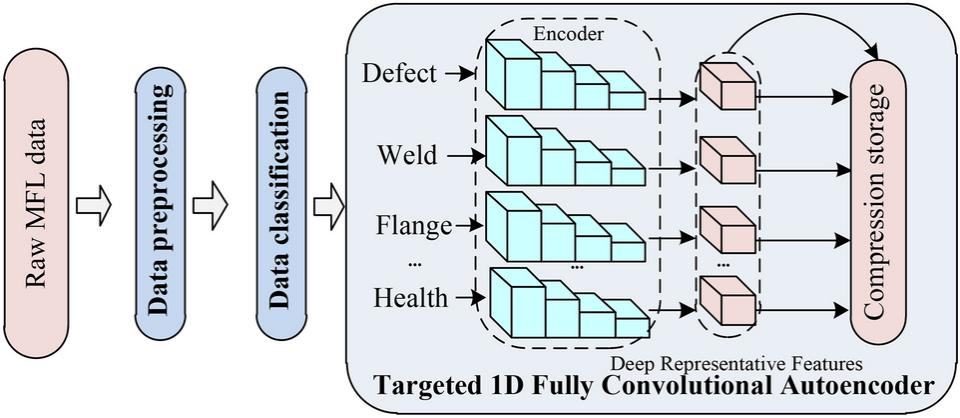
Intelligent compression scheme for MFL data.

As shown in Fig. 1, this paper presents the overall scheme for intelligent compression of MFL data. The scheme consists of three main parts: data preprocessing, data classification, and the construction of targeted one-dimensional (1D) fully convolutional autoencoder structure. The data preprocessing includes four steps: baseline correction, anomaly detection, data interpolation, and filtering. After preprocessing, the generated high-quality data is classified to distinguish different data types. Subsequently, based on the identified data types, targeted 1D fully convolutional autoencoder networks are constructed to effectively extract deep representative features specific to each data type. These features are then stored to achieve data dimensionality reduction compression. Detailed explanations of each part of the design method will be provided below.

### Data preprocessing

In the process of recording and storing data from the MFL inspection instrument for pipelines, the collected data is susceptible to anomalies and missing values due to the influence of sensors and environmental factors. Additionally, the data may be contaminated by various types of noise. To eliminate the impact of these anomalies and noise on subsequent data analysis, it is essential to perform data preprocessing on the collected MFL data^26^. Data preprocessing involves four steps: baseline correction, anomaly detection, data interpolation, and filtering.

First, the baseline correction aims to ensure that data with the same characteristics collected by different sensors have a consistent reference value, which is calculated as follows:

1 $$S^{\prime}_{i} = S_{i} - M(S_{i} ) + \frac{1}{C}\sum\limits_{i = 1}^{C} {M(S_{i} )} .$$

where *S*_*i*_ denotes the *i*-th channel MFL original signal, $M\left( \cdot \right)$ represents the median calculator, *C* denotes the total number of channels, and $S_{i}^{\prime }$ refers to the corrected *i*-th channel MFL signal.

The anomaly detection process is primarily aimed at segmenting the smooth and peak regions of anomalies within the data and recording their respective coordinates for subsequent interpolation.

Data interpolation is performed on the detected anomaly coordinates recorded during the anomaly detection phase, with the aim of repairing the anomaly amplitude at the anomaly coordinate points. Considering the execution time of the algorithm, linear interpolation method is used in this paper. Assuming that there is an anomalous point (*x*_*a*_,* y*_*a*_) between these two points (*x*_*0*_*, y*_*0*_) and (*x*_*1*_*, y*_*1*_), according to the linear equation established above, the anomalous magnitude *y*_*a*_ at this point can be interpolated back to the normal value *y*_*a*_ since *x*_*a*_ is known, which is calculated as follows:

2 $$y_{a} = \frac{{(x_{a} - x_{0} )}}{{\left( {x_{1} - x_{0} } \right)}}\left( {y_{1} - y_{0} } \right).$$

Filtering is applied to remove noise and small peak anomalies from the signal. In this study, the classical Gaussian filtering method is employed for processing, computed as shown in (3).

3 $${\mathbf{S}}^{*} = {\mathbf{S}}^{\prime } * \frac{1}{2\pi \sigma }e^{{ - \left( {x^{2} + y^{2} } \right)/2\sigma^{2} }}$$

where ${\mathbf{S}}^{*}$ is the filtered MFL data matrix, ${\mathbf{S}}^{\prime }$ is the interpolated MFL data matrix, and $\sigma$ refers to the standard deviation of the two-dimensional Gaussian kernel.

### Data classification

The common types of MFL detection curves mainly consist of three types of data^27^:

1. Pipeline defect data: At locations with pipeline defects such as corrosion, cracks, grooves, etc., the detection curve exhibits higher amplitudes.
2. Pipeline attachment data: At locations with pipeline attachments such as flanges, welds, branch pipes, valves, casings, etc., the detection curve also shows fluctuating variations, but this portion of the curve has a fixed pattern, making it easily distinguishable from defect data.
3. Healthy data: Apart from areas with defects and pipeline attachments, the majority of data forms relatively flat curves with minimal fluctuations, representing the healthy state of the pipeline wall.

As described above, different types of data exhibit significant differences in their level of fluctuations and manifestations. Autoencoder network structures are sensitive to data correlations, and they may have weaker generalization capabilities when dealing with two data groups with substantial differences, while performing well with data groups that have minor differences. Therefore, it is necessary to distinguish the data types at the input of the autoencoder structure to construct and train targeted autoencoder networks. Data classification involves several steps, such as segmentation, first-order differencing processing, energy and peak value calculations, etc. These steps are used to effectively differentiate data types based on their specific characteristic values. Thresholds are set accordingly to distinguish the data types effectively. The specific steps are as follows.

#### Data segmentation

**Fig. 2:**
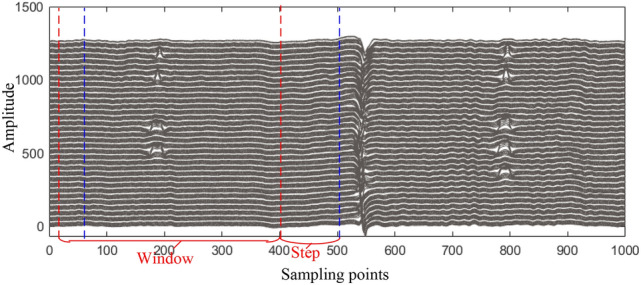
Schematic diagram of data block segmentation.

Data segmentation can effectively reduce the dimensionality of the model’s input data and facilitate data type categorization. As shown in Fig. 2, during the segmentation process, a sliding window $\Delta$ is set based on the characteristics of the MFL signal. The window has a length of *N*, a sliding step of $\eta$, and a default width of *C* channels for MFL data. By moving the *L* × *C* sliding window forward with a step size of* N*, multiple data blocks, denoted as *B*, are obtained. Subsequently, these data blocks *B* are processed and their data types are determined. On one hand, the density of features in the MFL signal is closely related to the data block length. On the other hand, the data block length potentially affects the parameters and computational cost of the autoencoder structure. Considering these two points, this study sets the window length to 400 to segment the data blocks.

#### First-order differential processing

Due to the strong correlation between the data detected by each probe of the MFL robot sensor, the differences between adjacent data points are relatively small. Therefore, after applying first-order differencing to the segmented data, the amplitude distribution range of the data becomes more concentrated. This concentration is advantageous for setting thresholds to differentiate data types^28^.

4 $$B^{\prime}_{i} (j) = B_{i} (j + 1) - B_{i} (j)$$

where $B_{i} ( \cdot )$ represents the *i*-th leakage data in data block *B* and *j* represents the *j*-th sampling point.

#### Data classification

**Fig. 3:**
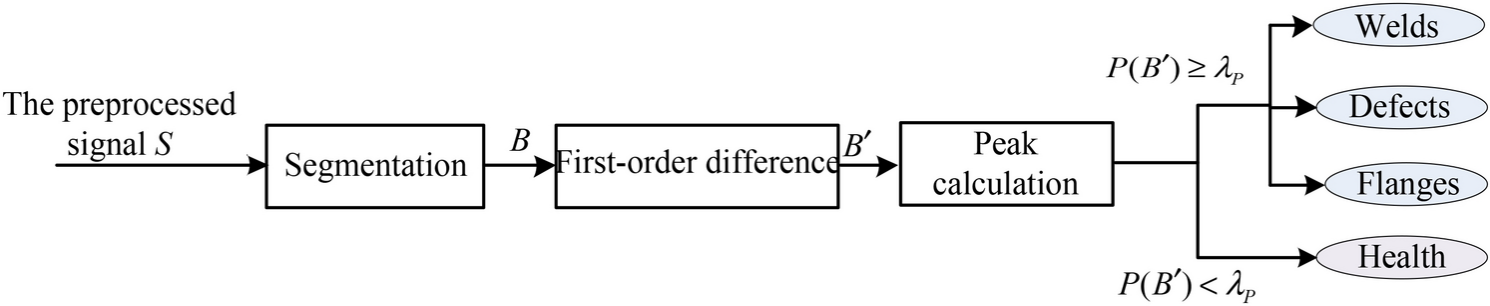
Overall flow of MFL data classification.

Due to the degree of signal fluctuation, differential pipeline component signals and defective signals typically exhibit higher signal peaks, while healthy signals have relatively smooth peaks. Therefore, based on an in-depth observation of the variation of the signal peaks, targeted thresholds can be set for the data block peaks of the weld, flange, defect, and health signals to finalize that data block type. The peak value of the data block is calculated in the following equation. The overall process of overall data categorization is shown in Fig. 3.

5 $$P(B^{\prime } ) = \sum\limits_{i = 1}^{C} {\left( {\max (B_{i}^{\prime } ) - \min (B_{i}^{\prime } )} \right)}$$

### Targeted one-dimensional fully convolutional autoencoder construction

As mentioned above, autoencoders are a type of network model that exhibits data correlations and demonstrates strong generalization performance on data with similar distributions. Therefore, in Section “Data classification”, a data classification process was designed to differentiate specific data types such as welds, defects, flanges, etc. Based on the distinguished data types, this section constructs targeted convolutional autoencoder models to effectively achieve data compression and dimensionality reduction.

#### Principle of the autoencoder

**Fig. 4:**
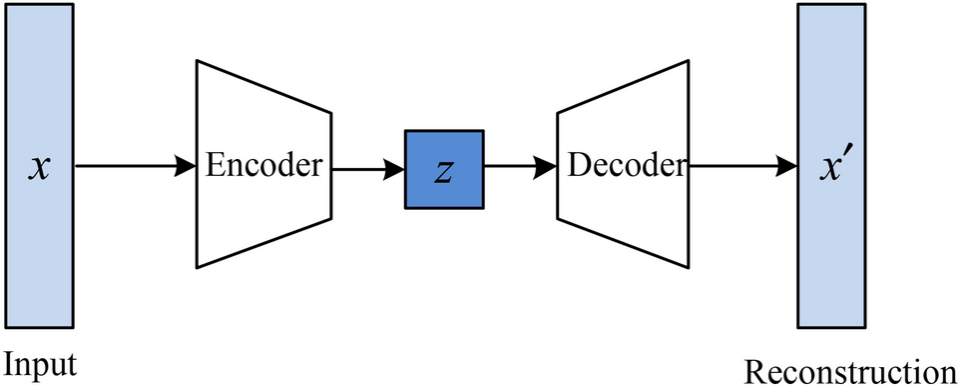
Conventional autoencoder structure.

The autoencoder is an unsupervised method for data dimensionality reduction and feature representation^29^. Its traditional structure is depicted in Fig. 4. It mainly consists of two parts: the encoder and the decoder. The encoder compresses the high-dimensional input *x* into a low-dimensional latent space representation *z*, while the decoder reconstructs this representation back into the output $x^{\prime}$. Therefore, the essence of an autoencoder is a data compression method, where both encoding and decoding are achieved through neural networks.

#### One-dimensional fully convolutional autoencoder (1D-FCAE) model construction

**Fig. 5.**
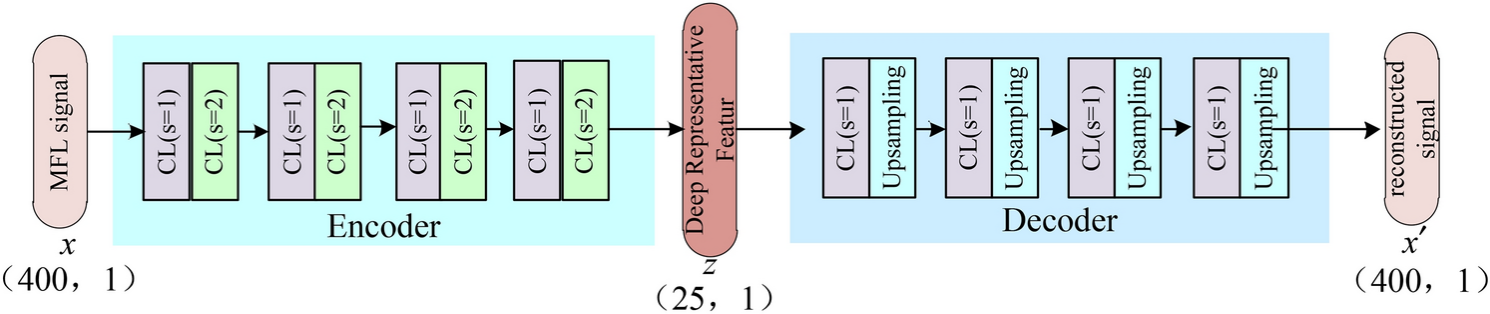
1D fully convolutional autoencoder model structure.

The convolutional autoencoder was introduced by researchers in 2016^29^, where convolutional layers and pooling layers were used instead of the original linear layers. This significantly reduced the model parameters, making the autoencoder model more lightweight and suitable for the industrial application. Considering the value and complexity of MFL data, ensuring effective deep feature extraction and high-precision reconstruction with the convolutional autoencoder model is challenging. To address this, this study improved the original structure by replacing pooling operations with convolutions with a stride of 2 to enhance the model’s learning capability. Subsequently, a 1D-FCAE model was proposed. The structure of the 1D-FCAE model designed in this study is illustrated in Fig. 5.

In Fig. 5, CL represents the Convolutional Layer followed by the Leaky Rectified Linear Unit (LeakyReLU) layer. Considering that the original pooling layers might weaken the model’s feature extraction capability, this study replaces the pooling layers with convolutional layers with a stride of 2 to achieve downsampling. Thus, in the encoder part, the feature extraction of the MFL signal is accomplished by stacking convolutional layers with strides of 1 (s = 1) and 2 (s = 2). This fully convolutional structure helps the encoder to learn the strong nonlinear relationship of the MFL signal and capture the intrinsic deep representation to accomplish the compression. In the decoder part, the channel size is adjusted using convolutional layers, and upsampling layers are utilized to restore the feature map’s size, ultimately achieving the reconstruction of the MFL signal. This convolution-based autoencoder design can effectively strengthen the model to learn the abstract representation of MFL and accomplish the compression and reconstruction.

As the decoder is the exact inverse operation of the encoder, this paper only presents the model parameters of the encoder, as shown in Table 1. From Table 1, it can be observed that the encoder consists of 8 CL modules, and through four downsampling operations using convolutions with a stride of 2, the original input signal dimension is reduced from (400, 1) to (25, 1). With stacked convolutional layers, the encoder can encode deep features of the MFL signal and achieve significant dimensionality reduction, which greatly reduces the storage space requirement, making it an effective data compression method. Additionally, the model has a small parameter size, making it easy to deploy on edge devices, and thus holds practical value.

**Table 1:** Encoder parameters of ID-FCAE.

Layer	(Channel, kernel_size, strides)	Param	Output shape
CL_1	(32, 3, 1)	128	(400, 32)
CL_2	(32, 3, 2)	3104	(200, 32)
CL_3	(16, 3, 1)	1552	(200, 16)
CL_4	(16, 3, 2)	784	(100, 16)
CL_5	(8, 3, 1)	392	(100, 8)
CL_6	(8, 3, 2)	200	(50, 8)
CL_7	(4, 3, 1)	100	(50, 4)
CL_8	(1, 3, 1)	13	(25, 1)

#### Model training parameter

During the training of the 1D-FCAE, an unsupervised approach is used to optimize the model, allowing it to learn the intrinsic deep representation of the MFL signal. The main training parameters of the model are presented in Table 2. In the forward pass, the reconstruction signal is computed using the training data and the model’s weight parameters. During the backward pass, the Mean Square Error (MSE) between the reconstruction signal and the input signal is calculated, and based on this, the Adam optimizer is used to optimize the weight parameters of the model’s layers. Through continuous training iterations, the model’s weight coefficients are gradually fine-tuned to adapt to different types of MFL signals. It is important to note that the targeted aspect in this study involves selecting different types of data for targeted parameter optimization of the 1D-FCAE. This allows the network’s layer parameters to adapt to specific types of data. During testing, specific data types are input into the targeted training encoder to obtain dimensionality-reduced compression features.

**Table 2:** ID-FCAE training parameters.

Optimizer	Loss function	Batch size	Epochs	Learning rate
Adam	MSE	16	200	0.001

The MSE calculation formula is as follows:

6 $$MSE = \frac{1}{m}\sum\limits_{i = 1}^{m} {\left( {x_{i} - x_{i}^{\prime } } \right)^{2} }$$

where $x_{i}$ is the input MFL signal, $x_{i}^{\prime }$ is the reconstructed signal, and *m* is the number of samples within a batch.

## Experimental validation

### Description of experimental data

**Figure. 6:**
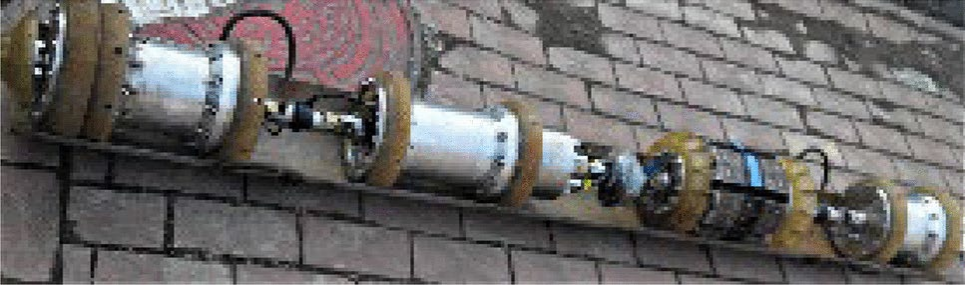
MFL internal detector.

**Fig. 7:**
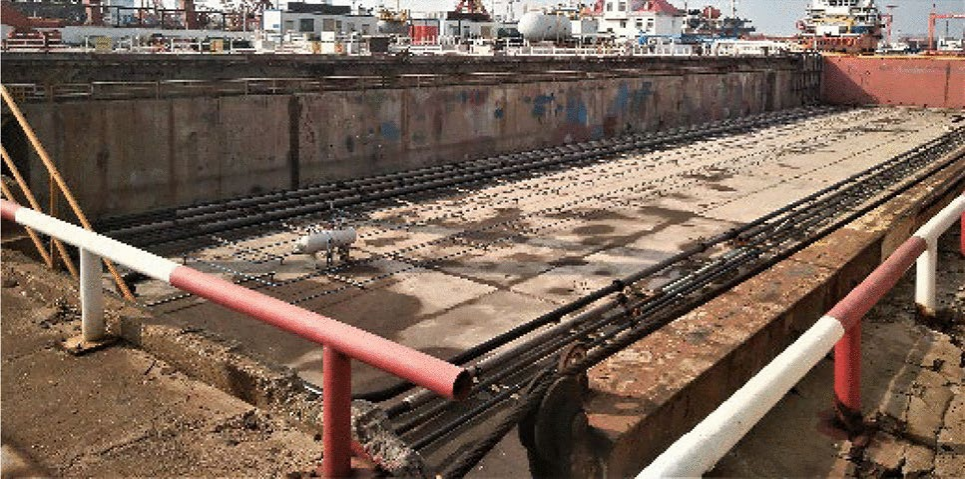
Experimental site.

The data used in this paper comes from actual measurements obtained from an experimental platform in northern China. Figure 6 shows the experimental site, and Fig. 7 shows the MFL inline inspection robot used. The pipeline section measured is approximately 50 m in length, with a diameter of 32 inches and is made of X65 carbon steel. The MFL inspection robot is equipped with 312 Hall sensors to ensure detection completeness. The sensor sampling frequency is one data point per 2 mm, so each sensor signal contains 25,000 sampling points, resulting in a final data matrix of 25,000 × 312. The collected signals include both healthy and defective signals, as well as characteristic signals from flanges and welds, making them suitable for comprehensive algorithm validation and analysis. In addition, most of the defects in the signal are corrosion, with only a very small number of cracks. The ratio of corrosion to cracks is approximately 0.95:0.05. The software environment used in this paper is based on the PyTorch framework, and the hardware environment consists of an NVIDIA GeForce RTX 3070 GPU (8 GB) and a 12th Gen Intel(R) Core(TM) i7-12700KF CPU.

### Evaluation metrics

The compression assessment metrics mainly include compression aspects and error aspects.

#### Compression aspects

In terms of compression, the compression performance of the algorithm is evaluated using indicators such as compression ratio (CR), compression rate, and compression time.

(1) Data Compression Ratio (CR) is an important performance metric for evaluating data compression techniques, and its calculation formula is as follows:

7 $$CR = \frac{{L_{in} }}{{L_{out} }}$$

where $L_{in}$ represents the number of bits in the input data stream, and $L_{out}$ represents the number of bits in the output data stream. When CR > 1, the data is compressed, and a higher CR value indicates better compression performance of the algorithm.

(2) Compression Rate is synonymous with Compression Ratio and is the reciprocal of the compression ratio. A smaller value indicates better compression performance.

(3) Compression Time refers to the execution time of the data compression algorithm. A shorter time represents higher algorithm efficiency.

#### Error aspects

The error refers to the discrepancy between the reconstructed signal after decompression and the original signal. A smaller error indicates higher accuracy of the compression algorithm, as it can preserve the essential features of the original signal to the maximum extent. In this study, Mean Absolute Error (MAE) and Root Mean Square Error (RMSE) are used for error evaluation, and their specific calculations are as follows.

8 $$MAE = \frac{1}{m}\sum\limits_{i = 1}^{m} {\left| {x_{i} - x_{i}^{\prime } } \right|}$$

where *m* represents the total number of channels in the MFL curve, $x_{i}$ denotes the original signal, and $x^{\prime}$ represents the reconstructed signal.

9 $$RMSE = \sqrt {\frac{1}{m}\sum\limits_{i = 1}^{m} {\left( {x_{i} - x_{i}^{\prime } } \right)^{2} } }$$

### Experimental analysis

#### Analysis of data preprocessing:

**Fig. 8:**
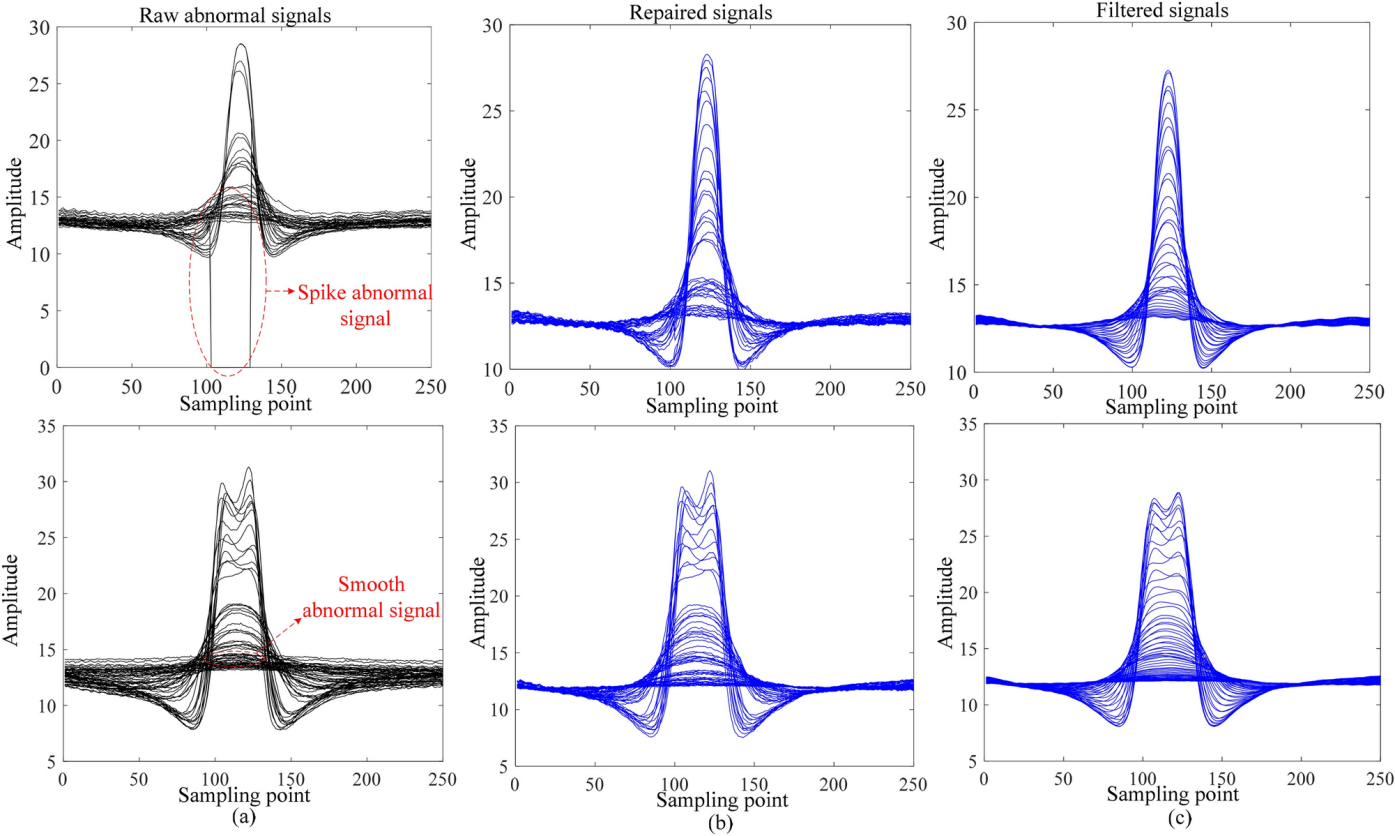
Comparison of MFL signal data before and after preprocessing. (**a**) Raw abnormal signal. (**b**) Repaired signal. (**c**) Filtered signal.

We selected typical MFL anomaly signals including smoothing and spiking anomalies, and utilized the data preprocessing process in Section “Overall scheme of MFL data compression” for signal repair and filtering, and the results are shown in Fig. 8. It can be observed in Fig. 8b that after data interpolation, the spikes and smoothing anomalies in the original anomalous signals are effectively repaired and the complete information of the signals is complemented, which is beneficial to the subsequent data classification and autoencoder training. In addition, by further performing Gaussian filtering on the repaired signal, the results are obtained as shown in Fig. 8c. As can be seen in Fig. 8c, the small burrs in the signal are effectively filtered out, which effectively improves the signal-to-noise ratio of the signal and facilitates the subsequent signal analysis.

In order to further verify the advantages of Gaussian filtering over other filtering methods, we choose the classical filtering methods including mean filtering and median filtering for comparison, and also utilize the peak signal-to-noise ratio (PSNR), signal-to-noise ratio (SNR) and distortion ratio (DR) metrics to compare the effectiveness, which are calculated as follows:

10 $$PSNR = 10 \cdot \lg \frac{{\left( {x_{\max } - \overline{x}} \right)^{2} }}{{s^{2} }}$$

where* s*^*2*^ is the variance of the signal, *x*_*max*_ is the maximum amplitude of the signal, and $\overline{x}$ is the mean of the signal.

11 $$SNR = \frac{{PSNR_{n} }}{{PSNR_{0} }}$$

where $PSNR_{n}$ is the peak signal-to-noise ratio after filtering and $PSNR_{0}$ is the peak signal-to-noise ratio before filtering. The higher the signal-to-noise improvement ratio, the better the denoising effect of the filtering method.

12 $$DR = \frac{{\left\| {x_{i}^{\prime } - x_{i} } \right\|}}{{\left\| {x_{i} } \right\|}}$$

where *x*_*i*_ and $x^{\prime}_{i}$ represent the collected signals before and after filtering, respectively, and $\left\| \cdot \right\|_{2}$ represents the L_2_ norm. The waveform distortion rate indicates the degree of damage caused by denoising on the useful waveform. The smaller this ratio, the closer the corrected waveform is to the original waveform, indicating less damage to the waveform and better filtering performance; conversely, a larger distortion rate (DR) indicates poorer correction performance.

The experimental results of the filtering comparison are shown in Table 3. In Table 3, it can be seen that the Gaussian filtering effectively improves the PSNR and SNIR of the signal with minimum DR compared to the mean and median filtering methods, which further indicates that the designed filtering method effectively filters out the noise from the signal while avoiding signal distortion.

**Table 3:** Performance comparison of different filtering methods.

	PSNR	SNIR	DR
Raw signal	9.0976	-	-
Gaussian filtering	**15.9687**	**1.755**	**0.031**
Mean filtering	15.7981	1.736	0.049
Median filtering	15.882	1.746	0.038

Significant values are in bold.

#### Analysis of data classification

**Fig. 9:**
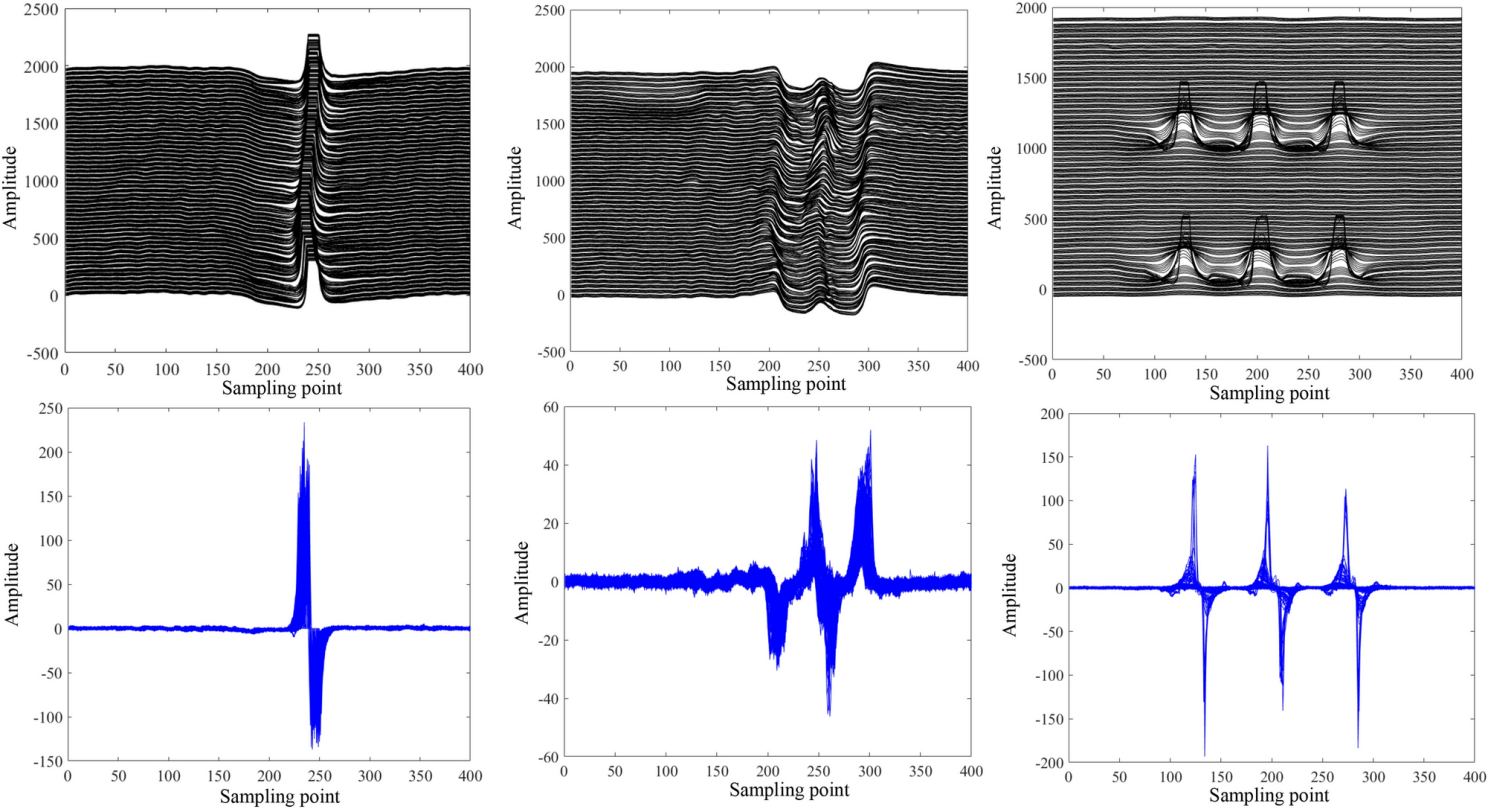
Comparison of signals before and after differential.

After the data segmentation, we can obtain about 246 data blocks, after that these data blocks are differentially processed and the results are shown in Fig. 9. In Fig. 9, we show the comparison between the original signal and the differential signal for typical welds, flanges and defects. As can be observed in Fig. 9, the amplitudes become more concentrated and distinguishable after the differential processing, which facilitates the setting of targeted thresholds for data block classification. Specifically, the maximum amplitude of the differential weld signal is between 200 and 250, the flange signal amplitude is between 40 and 60, the defect signal amplitude is between 150 and 200, and the amplitude of the health signal is near 0. Based on this, this study sets the classification thresholds for the weld, flange, and defect signals to 200, 150, and 40, respectively, with the remainder being the healthy signal.

#### Analysis of ID-FCAE reconstruction effectiveness

**Fig. 10:**
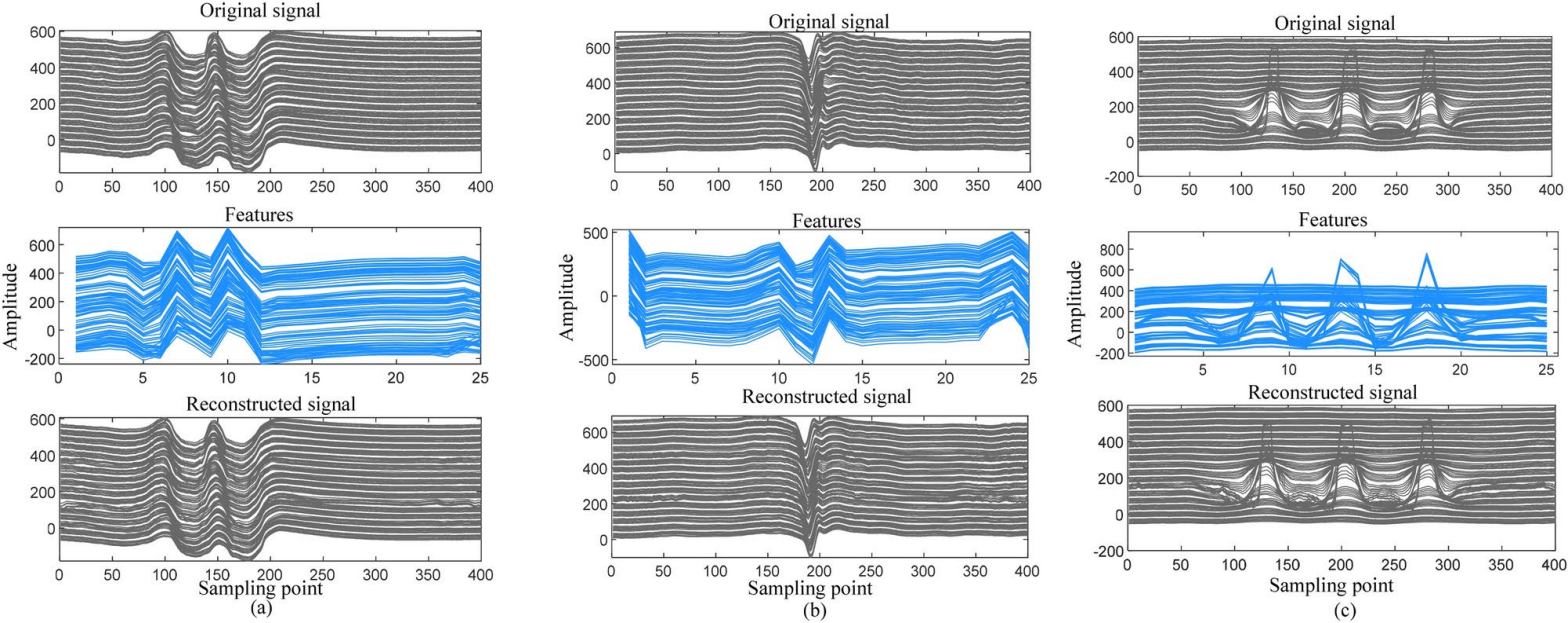
Different signal reconstruction effects of ID-FCAE model. (**a**) Flange signal. (**b**) Weld signal. (**c**) Defect signal.

In this section, targeted ID-FCAE training and testing are conducted based on the data block classification results. Considering the significant amplitude variations and high complexity of signals from pipeline features and defects, which make accurate signal reconstruction challenging, this study focuses on training and testing ID-FCAE specifically for three complex signals: flanges, welds, and defects. The original, feature, and reconstructed signals are illustrated in Fig. 10.

From Fig. 10, it can be observed that after the original signal is downsampled and compressed by the encoder, the deep representations of different types of signals can be obtained and the representations can effectively reflect the contour and detail information of the MFL signals, which suggests that the full convolutional structure in the encoder can encode the intrinsic deep representations of the MFL signals. This helps to achieve MFL signal compression and facilitates the decoder to achieve fine-grained reconstruction of the original signal. In addition, according to the reconstructed signals, it can be seen that the decoder is able to accurately restore the key details of the original signals, realize fine-grained signal reconstruction, and effectively retain the basic feature information of the original signals. This verifies that ID-FCAE has strong compression and reconstruction capabilities.

**Fig. 11:**
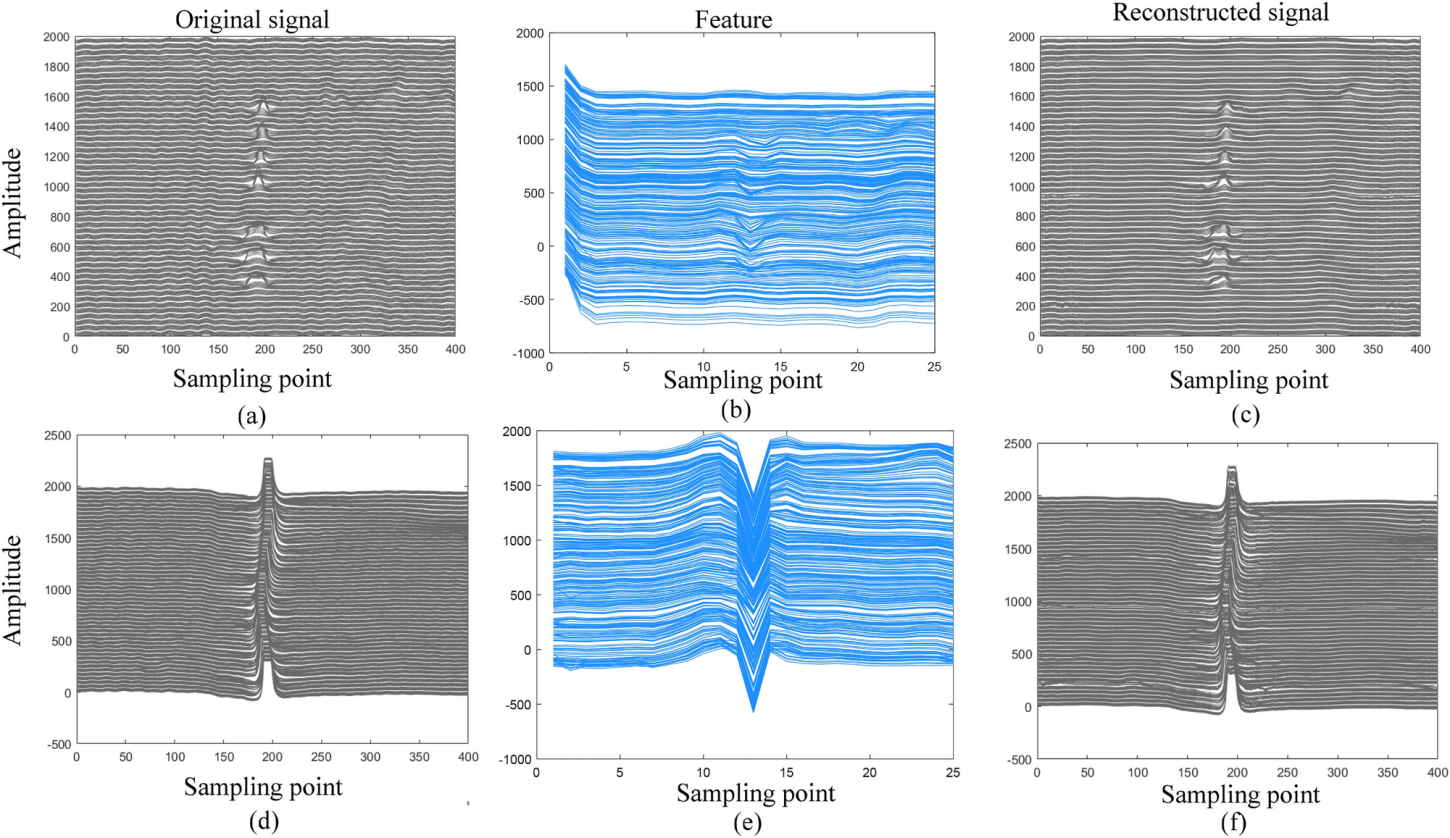
Reconstruction generalizability test using 12-inch pipeline MFL signals.

In addition, we conducted a generalizability test of the reconstruction using the MFL signal of a 12-inch pipe, which contains more complex defect patterns, and the results are shown in Fig. 11. As can be seen in Fig. 11, the proposed method can still achieve accurate reconstruction of MFL signals and restore the characteristic details of complex defects, which further demonstrates that 1D-FCAE possesses strong generalization and applicability.

In addition, we choose the classical unsupervised dimensionality reduction method PCA and the original autoencoder (AE) to perform comparative experiments utilizing MFL signals from 32-inch and 12-inch pipes, and the results are shown in Table 4. For the 32-inch dataset, the proposed method significantly improves the reconstruction accuracy on different types of signals. Taking the flange signal as an example, the proposed ID-FCAE in this paper reduces the MAE by about 21% and the RMSE by about 28% compared with PCA, which indicates that the proposed method is able to learn the intrinsic deep representation of the complex MFL signals and accurately restore the important details of the original signals compared with the traditional methods. In addition, compared with the autoencoder method, 1D-FCAE reduces the MAE and RMSE by 13.4% and 21%, respectively, which indicates that the introduction of convolution effectively strengthens the feature extraction capability of the encoder and contributes to a finer reconstruction.

**Table 4:** Results of PCA and 1D-FCAE comparison experiments.

Datasets	Methods	Error	Flange	Weld	Defect
32-inch MFL signal	PCA	MAE	5.22	3.44	2.37
		RMSE	3.15	5.63	5.75
	1D-AE	MAE	4.52	2.92	1.95
		RMSE	2.87	5.34	4.99
	ID-FCAE	MAE	**4.12**	**2.71**	**1.40**
		RMSE	**2.27**	**4.97**	**4.65**
12-inch MFL signal	PCA	MAE	6.32	4.37	2.94
		RMSE	4.23	6.53	6.54
	1D-AE	MAE	5.62	4.09	2.38
		RMSE	3.89	5.98	5.72
	ID-FCAE	MAE	**5.14**	**3.02**	**1.85**
		RMSE	**3.25**	**5.16**	**5.33**

Significant values are in bold.

For the 12-inch dataset, due to the presence of more complex defect patterns, 1D-FCAE still has the most superior reconstruction accuracy with a slight decrease in accuracy, which indicates that the proposed method possesses generalizability and scalability over different pipeline MFL signals, facilitating its practical application.

#### Analysis of 1D-FCAE computational cost

We further evaluate the computational cost of the method using parameters, memory usage and training time, and the results are shown in Table 5. From Table 5, we can observe that compared with the traditional AE structure utilizing fully connected layers, the proposed 1D-FCAE reduces the parameters, memory usage and training time by 206.8 k, 1.58G and 80 s, respectively, which suggests that the fully convolutive structure effectively reduces the computational cost of the model, making it suitable for application in resource-limited industrial environments.

**Table 5:** Comparison of computational costs.

Methods	Parameter (k)	Memory usage (G)	Training time (s)
1D-AE	213.628	2.36	130
ID-FCAE	6.842	0.781	50

#### Analysis of ID-FCAE compression performance

In order to compare the compression performance and compression efficiency of the algorithms, classical compression methods like LZW and Huffman, shallow machine learning methods such as PCA and deep learning methods including 1D-AE are selected for comparison in this study respectively and the experimental results are shown in Table 6. In order to compare the compression performance and compression efficiency of the algorithms, classical compression methods like LZW and Huffman, shallow machine learning methods such as PCA and deep learning methods including 1D-AE are selected for comparison in this study respectively and the experimental results are shown in Table 6. As shown in Table 6, the proposed ID-FCAE method improves the compression rate by about 33% compared to LZW coding. In addition, the compression time is reduced by about 48% and the decompression time is reduced by about 53%. The compression rate of the proposed ID-FCAE method is improved by about 14% compared to the PCA method. In addition, the compression time is reduced by about 34% and the decompression time is reduced by about 43%. These results fully demonstrate that the proposed method can effectively improve the compression rate and compression efficiency compared with the traditional method, which helps lossless storage in practice.

**Table 6:** Comparative analysis of conventional methods and ID-FCAE compression experiment.

Method	Compression ratio	Compression rate	Compression time (ms)	Decompression time (ms)
Huffman coding	6.66: 1	0.15	423	786
LZW coding	12: 1	0.08	267	394
PCA	14: 1	0.07	212	326
1D-AE	16: 1	0.06	200	256
ID-FCAE	**16:1**	**0.06**	**140**	**187**

Significant values are in bold.

In addition, although the compression rate of 1D-FCAE is consistent with that of this paper, its compression and decompression time is significantly higher than that of the method in this paper. This is due to the fact that 1D-FCAE significantly reduces the parameters and computation by introducing the convolution operation, which infuses the proposed method with lightweight attributes and makes it easy to deploy the application in practice.

## Conclusion

This paper proposes an intelligent data compression method based on targeted ID-FCAE for MFL non-destructive testing in long-distance oil and gas pipelines, which generates a large amount of data. Based on the characteristics of MFL data, a data preprocessing and data block classification method is designed. Data preprocessing effectively performs operations such as original data correction, interpolation, and filtering, generating high-quality data for subsequent processing. Moreover, by setting targeted thresholds, different data block types can be effectively distinguished. For different data block types, a targeted ID-FCAE intelligent dimensionality reduction compression method is specifically designed. Experimental validation shows that this method has strong feature extraction capabilities and high reconstruction accuracy, enabling fine-grained signal reconstruction while achieving dimensionality reduction compression. Through practical experimental analysis, the reconstruction error such as MAE is reduced by about 27.7% and the compression ratio is improved by about 14% compared with traditional methods such as PCA. In addition, the proposed 1D-FCAE reduces 206.8 k, 1.58G, and 80 s in parameters, memory usage, and training time, respectively, and reduces compression and decompression time by 60 and 69 ms, respectively, compared to ID-AE. These improvements fully demonstrate the proposed method possesses superior compression performance and is easy to be applied in industrial environments.

However, the proposed method still has limitations. First, data classification relies on manual threshold tuning, so an adaptive thresholding method needs to be investigated for further optimization. In addition, the proposed method may have limited generalization for large-scale MFL signals with mixed modes, so how to strengthen the feature extraction capability and self-evolutionary nature of the autoencoder to enhance the generalization of the model also needs to be further studied in the future.

## Data Availability

Data are available on request from the authors. If you have any request, please contact xuanwb@pipechina.com.cn.
